# A Rare Case of Triple-Positive Breast Cancer With Eventual Triple-Negative Small Bowel Metastasis

**DOI:** 10.7759/cureus.74308

**Published:** 2024-11-23

**Authors:** Micah Ngatuvai, Anthony Pasarin, Abanoub Gabra, Ihor Pidhorecky

**Affiliations:** 1 Orthopedics, Nova Southeastern University Dr. Kiran C. Patel College of Allopathic Medicine, Fort Lauderdale, USA; 2 Orthopedics, Texas Tech University Health Sciences Center, El Paso, El Paso, USA; 3 Orthopedics, William Beaumont Army Medical Center, El Paso, USA; 4 Surgical Oncology, HCA Florida Westside Hospital, Plantation, USA; 5 Pathology, HCA Florida Westside Hospital, Plantation, USA

**Keywords:** carcinoembryonic antigen, estrogen-receptor, human epidermal growth factor receptor-2, invasive lobular carcinoma, progesterone-receptor, small bowel

## Abstract

Invasive lobular breast cancer (ILBC) is a common cause of breast cancer. Prognosis is dependent on many factors such as metastasis location and hormone receptor positivity. A 59-year-old postmenopausal African-American female who was referred to our clinic in May of 2022 presented with a suspicious small bowel lesion seen on surveillance imaging. The patient was diagnosed 15 years prior, with hormone receptor triple positive ILBC of the left breast, T1N2M0. In March of 2021, the patient was admitted to the hospital for rectal bleeding and was also found to have an elevated carcinoembryonic antigen (CEA) level. Computed tomography of the abdomen and pelvis was performed, which revealed a 4 cm segment of proximal ileum that was indeterminate for inflammation, neoplasm, or focal ischemia. The patient ultimately agreed to proceed with a diagnostic laparoscopy to further identify this area and underwent a small bowel resection. The final pathology of this specimen revealed a poorly differentiated, diffuse-type carcinoma with a focal mucinous matrix. The tumor involved the submucosa, serosa, and pericolonic adipose tissue. The morphology was consistent with metastatic carcinoma originating from ILBC. The hormone receptors for this specimen revealed estrogen-receptor (ER) negative, progesterone-receptor (PR) negative, and human epidermal growth factor receptor-2 (HER-2) negative. Lobular breast cancer metastasis to the small bowel as well as triple positive to triple negative conversion is exceedingly rare. The recurrence and metastasis of breast cancer are considerably high and the survival rate for these individuals has shown little improvement throughout the last decade. Defining relevant prognostic markers is crucial for improved management with known metastasis and triple negative receptors.

## Introduction

Breast cancer is one of the most prevalent cancers among women in the world [1]. The two main subtypes of breast cancer include invasive ductal breast cancer (IDBC) and invasive lobular breast cancer (ILBC). In particular, ILBC accounts for 5-15% of all mammary carcinomas [2]. Women who receive an early diagnosis and timely treatment of breast cancer have a substantially improved chance of survival [3]. Despite early detection, there is an approximately 30% possibility of cancer recurrence and metastasis during a patient's lifetime [3]. Metastasis is the leading cause of mortality among breast cancer patients [4]. Common metastatic sites of IDBC and ILBC breast cancer include the intra-abdominal viscera, uterus, ovaries, peritoneum, and retroperitoneal surfaces. Involvement of the gastrointestinal (GI) tract, specifically, the small bowel, is incredibly rare [5]. In the literature, there are few reported cases worldwide of primary breast cancer metastasizing to the small bowel, more specifically that present with a complete conversion of hormonal status [6]. Additionally, cases of hormonal triple-negative breast cancer (estrogen-receptor (ER), progesterone-receptor (PR), and human epidermal growth factor receptor-2 (HER-2)) are seen in only 10% of breast cancers [7]. This report of a rare case describes an initial diagnosis of triple-positive breast cancer, with a recurrence as triple-negative breast cancer that metastasized to the small bowel 15 years post-treatment.

## Case presentation

A 59-year-old postmenopausal African-American female was referred to our clinic in May 2022 and presented with a suspicious small bowel lesion seen on surveillance imaging. The patient was diagnosed 15 years prior with ILBC of the left breast, T1N2M0, with no other history of neoplastic disease. At that time, she had two separate tumors measuring 1.5 cm each with both tumors having triple-positive (ER, PR, HER-2) hormonal receptor status. After the initial diagnosis, the patient received a timely treatment of neoadjuvant chemotherapy with Taxotere, carboplatin, and Herceptin, followed by a left simple mastectomy with an additional one year of adjuvant Herceptin.

In March 2021, the patient was admitted to the hospital for rectal bleeding and was found to have an elevated carcinoembryonic antigen (CEA). The patient received a computed tomography (CT) scan, positron emission tomography (PET), and a colonoscopy, which combined showed unremarkable findings. Of note, the ileoscopy could not examine the abnormal ileal site noted from the prior imaging. The patient was also advised to undergo a balloon enteroscopy to further examine the small bowel, which was not performed until April 2022.

**Figure 1:**
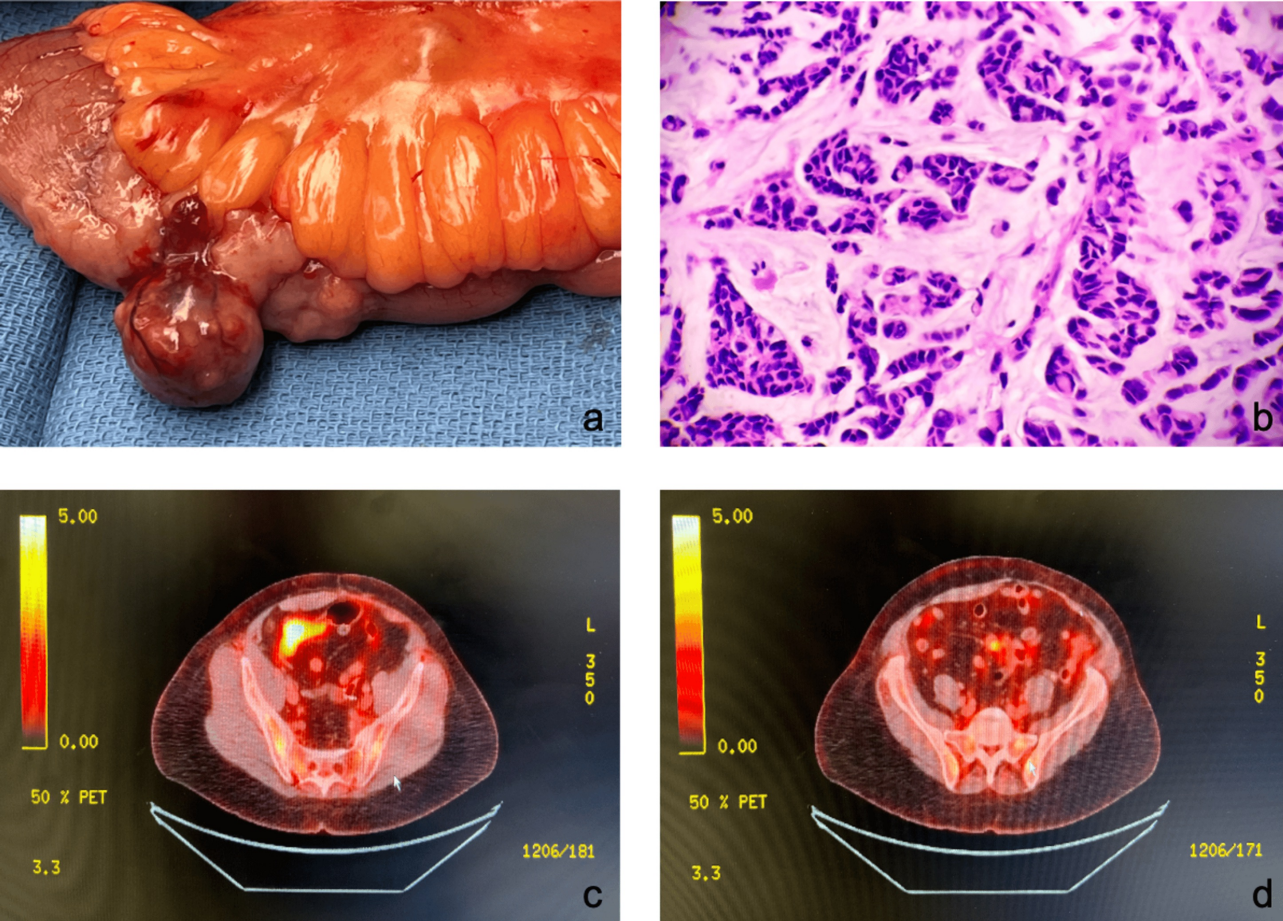
Breast cancer metastasis to small bowel (a) Gross image of small bowel surgical specimen containing exophytic mass. (b) Hematoxylin and eosin stain of invasive lobular carcinoma demonstrating classic “Indian File” of neoplastic cells. (c) Initial PET-CT axial view revealing increased metabolic activity in the loop of the ileum. (d) Initial PET-CT axial view revealing increased metabolic activity in multiple mesenteric lymph nodes. PET: positron emission tomography; CT: computed tomography

The patient was sent to our clinic in April 2022 with a report of the endoscopy with biopsy, which was unremarkable, and lab results showing an elevated CEA. A mammogram was performed and showed a 6-mm lobulated mass in the right breast for which a biopsy was recommended. The right breast biopsy was found to be negative for atypical hyperplasia or malignancy. A magnetic resonance imaging enterography and PET scan were subsequently performed and compared to the imaging from 2021. The results from the MRI showed a persistent segment of abnormal wall thickening involving an ileal loop in the right mid-abdomen with adjacent mild mesenteric adenopathy (Figure 1d). The PET-CT scan showed increased hypermetabolic segmental wall thickening involving the ileal loop in the right abdomen (Figure 1c).

In June of 2022, the patient underwent a diagnostic laparoscopy with small bowel resection due to suspected metastatic lobular breast cancer in the small bowel. During the procedure, in the mid-ileum, a pedunculated tumor with nearby lymphadenopathy was identified. A portion of the small intestine with attached mesentery was resected. The tumor measured 3.1 x 1.9 x 1.9 cm and was yellow-pink in color and solid, as seen in Figure 1a.

According to a histopathological examination, the portion of the bowel resected showed poorly differentiated diffuse-type carcinoma with a focal mucinous matrix. The tumor involved the submucosa, serosa, and pericolonic adipose tissue. The morphology was most consistent with metastatic carcinoma originating from ILBC. Histologically, the tumor cells were discohesive with an infiltrative pattern through the minimal stromal reaction. The cells were arranged in nests, trabeculae, and rare single-cell infiltration, with the classic "Indian-file" arrangement (Figure 1b). Cytological atypia revealed a high nuclear-to-cytoplasmic ratio, with pleomorphic nuclei and hyperchromasia. The tumor cells were positive for GATA-3, which proves mammary origin, and negative for E-cadherin, which proves lobular carcinoma type. The Ki67 index was 90%. Sixteen of the total sixteen lymph nodes obtained were found to be positive for metastasis. The hormone receptors were ER-negative (0%), PR-negative (0%), and HER-2 not overexpressed (0%).

The patient had no surgical complications and remained in the hospital for a few postoperative days. The patient was placed on a chemotherapy regimen consisting of pembrolizumab and Taxol. Regular follow-up with a medical oncologist was scheduled to monitor her clinical course.

## Discussion

In the present case, the patient was treated for triple-positive ILBC and, over the course of 15 years, developed conversion to triple-negative status with metastasis to the small bowel. In a large study that analyzed more than 2,500 cases of breast cancer with metastatic disease over a period of 18 years, it found that only 17 patients (less than 1%) had metastasis to the GI tract, which highlights the rarity of small bowel metastasis [5]. Unfortunately, the mechanism of metastasis to the small bowel is unknown. Furthermore, GI metastasis is generally a sign of disseminated disease and carries a poor prognosis [8]. In fact, one such review found that breast cancer with metastasis to the small bowel carried a one-year survival of less than 50% [9].

Immunohistochemistry is a technique that allows for the identification of specific proteins in tissue samples, and it plays a crucial role in diagnosing and understanding the progression of breast cancer, as well as selecting the appropriate treatment options. Furthermore, hormone receptor expression also plays an important role in determining the prognosis as well as the treatment [10]. In the initial presentation, our patient was found to have a positive expression of ER, PR, and HER-2, which carries a relatively favorable prognosis [11]. Comparatively, patients who are triple-negative have a significantly shorter length of survival [12]. Unfortunately, the receptor expression for the recurrence of ILBC found in this patient was triple-negative, which carries a poor prognosis. Similarly, instances of receptor discordance in which the primary cancer is positive and metastasis is negative have also been shown to result in a significantly worse outcome [13]. In one study that reviewed how receptor conversion in primary breast cancer impacts outcomes, Zhao et al. found that approximately 20% of breast cancer patients had different molecular subtypes between primary and metastatic types [14]. In their study, the conversion rates among ER, PR, and HER-2 from the primary tumor to the earliest point in metastasis were 21.1%, 33.2%, and 11.6%, respectively [14]. Thus, the literature indicates that hormone receptor status can change over a period of time and after certain treatments have been initiated.

Breast cancer survival after metastasis is poor. The five-year survival for metastatic breast cancer in women is 29% [15]. The rate is worse in men, with only 22% surviving after a five-year period. Regardless, many treatment options are still available [15]. Lopez-Tarruella et al. found that primary tumor resection regardless of metastasis location, receptor expression, or tumor size was found to improve overall survival [16]. Chemotherapy regimens are also beneficial, with drugs such as pembrolizumab and Taxol, as used in our patient, which have been shown to significantly improve survival outcomes.

Current recommendations for screening for breast cancer recurrence involve the utilization of radiographic and clinical measures. Mammography is the primary radiographic surveillance tool used in women over the age of 30, which has been responsible for finding 8-50% of ipsilateral recurrences [17]. Many societies such as the American Society of Clinical Oncology recommend annual mammograms to monitor breast cancer recurrence [18]. Ongoing research is investigating ways to improve current mammogram techniques, such as the Tomosynthesis Mammographic Imaging Screening Trial (TMIST) funded by the National Cancer Institute (NCI). The trial aims to compare the number of advanced cancers detected in women screened for five years with 3D mammography to the number detected in women screened with 2D mammography [19]. Additionally, utilization of tumor markers, bone scans, CT scans, MRI, and PET scans may be beneficial when indicated. Moreover, regular follow-up with an oncologist is also important to monitor for new symptoms, gather an in-depth history, and perform a comprehensive physical exam. The National Comprehensive Cancer Center Network recommends a routine history and physical occurrence every four to six months for the first five years after primary therapy and annually thereafter [20].

The implications of this case for future research lie in the need to better understand metastasis mechanisms, receptor conversion, and the development of novel therapeutic strategies for patients with metastatic breast cancer. As the case highlights the rarity of small bowel metastasis and the poor prognosis of receptor discordance, further studies should focus on identifying the underlying factors and molecular pathways involved in these phenomena. Additionally, investigating the potential benefits of targeted therapies and personalized treatment approaches based on patients' molecular profiles may lead to improved survival outcomes and better management of the disease. Future research should also focus on improving electronic medical record communication between healthcare systems to facilitate more comprehensive and seamless longitudinal research. By analyzing similar cases and conducting in-depth research on the complex interplay between tumor biology, receptor status, and metastatic patterns, we can contribute to the advancement of breast cancer treatment and the overall understanding of this multifaceted disease.

## Conclusions

We report an extremely rare case of a patient with an initial diagnosis of triple-positive ILBC, 15-years post-treatment recurrence of triple-negative breast cancer metastasized to the small bowel. This case underscores the unpredictable nature of breast cancer evolution, emphasizing the importance of comprehensive monitoring and personalized approaches in patient care. Continual study of metastasis mechanisms, risk factors, and screening methods are needed in order to improve patient outcomes. Furthermore, the observed changes in receptor status and their direct impact on prognosis underscore the imperative to delve deeper into research on tailored treatments and to address the inherent challenges this variability presents.
